# Supplementary material for: Evidence supported by Mendelian randomization: impact on inflammatory factors in knee osteoarthritis
Source: Front Med (Lausanne). 2024 May 28;11:1382836. doi: 10.3389/fmed.2024.1382836 (PMC11165061; doi:10.3389/fmed.2024.1382836)
Supplement: Supplementary file 1 [file Data_Sheet_1.ZIP › data/GCST90274839_5e6/again/839.leaveoneout.pdf]

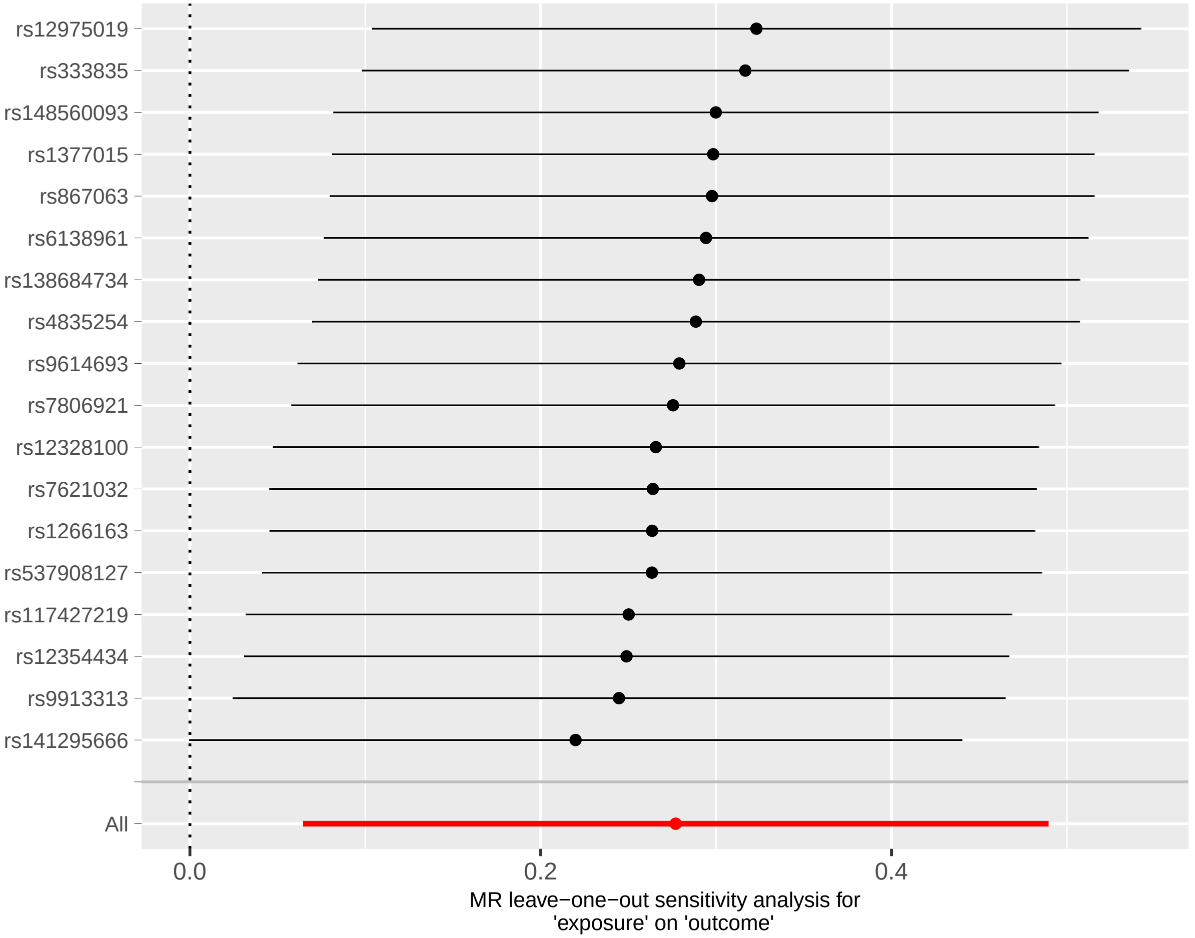

rs12975019
rs333835
rs148560093
rs1377015
rs867063
rs6138961
rs138684734
rs4835254
rs9614693
rs7806921
rs12328100
rs7621032
rs1266163
rs537908127
rs117427219
rs12354434
rs9913313
rs141295666
All
0.0
0.2
0.4
MR leave−one−out sensitivity analysis for
'exposure' on 'outcome'
